# Human Cytomegalovirus (CMV) Infection Associated With Decreased Risk of Bronchogenic Carcinoma: Understanding How a Previous CMV Infection Leads to an Enhanced Immune Response Against Malignancy

**DOI:** 10.7759/cureus.37265

**Published:** 2023-04-07

**Authors:** Selena Rashid, Amalia Ardeljan, Lexi R Frankel, Matthew Cardeiro, Enoch Kim, Brittany M Nagel, Kazuaki Takabe, Omar Rashid

**Affiliations:** 1 Department of Surgery, Michael and Dianne Bienes Comprehensive Cancer Center, Holy Cross Health, Fort Lauderdale, USA; 2 Department of Allopathic Medicine, Nova Southeastern University, Dr. Kiran C. Patel College of Allopathic Medicine, Fort Lauderdale, USA; 3 Department of Surgical Oncology, Roswell Park Comprehensive Cancer Center, Buffalo, USA; 4 Department of Surgery, University at Buffalo Jacobs School of Medicine and Biomedical Sciences, The State University of New York, Buffalo, USA; 5 Department of Medicine, University of Miami, Leonard Miami School of Medicine, Miami, USA; 6 Department of Surgical Oncology, Massachusetts General Hospital, Boston, USA; 7 Department of Surgical Oncology, Broward Health, Fort Lauderdale, USA; 8 Department of Complex General Surgical Oncology and General Surgery, Topline MD Alliance, Fort Lauderdale, USA; 9 Department of Surgical Oncology, Memorial Healthcare, Pembroke Pines, USA; 10 Department of Surgical Oncology, Delray Medical Center, Delray, USA

**Keywords:** oncology, microbiology, immunology, bronchogenic carcinoma, malignancy

## Abstract

Introduction:  ​Cytomegalovirus (CMV) causes a long-lasting, asymptomatic infection that reportedly has both advantageous and deleterious effects on tumor progression. The purpose of this study was to evaluate the correlation between CMV infection and the incidence of bronchogenic carcinoma.

Methods: The study was conducted using a Health Insurance Portability and Accountability Act (HIPAA) compliant national database to identify patients both with and without histories of CMV infection using International Classification of Diseases (ICD-10 and ICD-9) codes. Access to the database was granted by Holy Cross Health, Fort Lauderdale for the purpose of academic research with standard statistical methods used to analyze the data. 14,319 patients were included in both the control and CMV-exposed groups and matched by age range and Charlson Comorbidity Index (CCI) scores.

Results: The incidence of bronchogenic carcinoma was 1.69% (243/14,319 patients) in the CMV group and 6.08% (871/14,319 patients) in the control group. The difference was statistically significant by a p-value of less than 2.6x10^-16^ with an odds ratio of 0.26 (95% CI: 0.24-0.30). The two groups were also matched for treatment. Further evaluation of the CMV-specific treatment effects on outcomes was limited due to the insufficient number of treated patients in the control group.

Conclusion: This study found a statistically significant correlation between a prior CMV infection and a reduced incidence of bronchogenic carcinoma. This study demonstrates the need for further investigation into how the tumor microenvironment and host immune system are altered by the presence of a latent CMV infection.

## Introduction

Human cytomegalovirus (CMV) induces a long-lasting infection that has both advantageous and deleterious effects on tumor progression [1]. Viruses such as CMV have a predilection for the immune system and can remain latent within cells, conferring immune-modulating characteristics against certain malignancies [2]. Additionally, it has been shown that the presence of anti-CMV T cells can be indicative of decreased risk of developing malignancy [2]. It is possible that the massive proliferation of effector memory T cells allows for the retention to effectively kill immune targets [2]. Moreover, the reactivation of latent CMV infection may also be associated with enhanced natural killer cell activity, leading to cytotoxic effects against malignant tumor cells [3]. In mice, a CMV infection has been found to heighten the inflammasome-dependent release of Interleukin-18 (IL-18), further strengthening natural killer cell activity [4]. CMV infection has also been shown to increase the quantity of Cluster of Differentiation-8+ (CD8+) T cells during its clinical course, allowing for enhanced immune responses [5]. A latent CMV infection may provide stimulation of the immune system resulting in higher quantities of stimulated T cells, in turn, creating a stronger anti-tumor effect within individuals affected [5]. Additionally, viral infections with CMV require the participation of host cells' pattern recognition receptors, which are receptors that are responsible for recognizing the unique molecules of certain pathogens. This results in a massive release of cytokines and type 1 interferons [4].

The literature has also demonstrated that the immune system changes of a CMV infection may provide information that can be later utilized as an adjuvant therapy with immune checkpoint inhibitors. This can enhance the body's immune response to certain malignancies [6]. Although much of the literature provides details regarding CMV’s relationship to hematologic cancers such as leukemia and lymphoma, our findings provide substantial evidence suggesting that CMV infection confers significant survival and a later age of diagnosis in patients with bronchogenic carcinoma.

Bronchogenic carcinoma is a malignancy of lung tissue that has been one of the highest causes of death due to cancer worldwide [7]. CMV offers a protective effect against this malignancy due to the potential mechanisms in which this infection alters the tumor microenvironment. An active infection has been shown to have anti-tumor effects of recruiting inflammatory cytokines, increasing immune infiltration, and enhancing overall tumor killing [2]. These characteristics of CMV provide potential therapeutic benefits in the context of bronchogenic carcinoma cancer protection, as well as to the scientific community due to the relatively poor survival rates of patients with bronchogenic carcinoma. Through a literature search, it was found that prior studies have not detailed the role of CMV in conferring enhanced protection against this specific malignancy. In contrast, most of the literature has instead focused on the harmful immunological effects of a CMV infection and how it may increase the risk of neoplasm [1]. For these reasons, the purpose of this project was to investigate the protective mechanisms of a latent vs asymptomatic CMV infection on the development of bronchogenic carcinoma. This was primarily done to gain valuable insight that may provide strategies for enhancing certain immune mechanisms that can be used to prevent bronchogenic carcinoma development. 

This article was previously presented as a meeting oral presentation at the 2022 Academic Surgical Congress on February 3, 2022 and also as an abstract at the Sixth International Conference on Cancer Research & Drug Development on October 25-28, 2021.

## Materials and methods

Access to a Humana Health Insurance Portability and Accountability Act (HIPAA) compliant national database used for gathering data was offered by Holy Cross Health, Fort Lauderdale, Florida, for the express purpose of academic research. Using the International Classification of Disease 9th and 10th Codes (ICD-9, ICD-10) for CMV and bronchogenic carcinoma, a retrospective analysis from (January 2010 to December 2019) was performed. Based on the history of prior CMV infection, patients were designated to one of the two groups: the experimental or CMV-exposed group and the control group. These groups were then matched according to age, sex, and Charlson Comorbidity Index (CCI). A diagnosis of bronchogenic carcinoma was the primary outcome measure of this study. Results were analyzed using standard statistical methods, and relative risk was also used to estimate the odds ratio. Subsequent analysis of the data involved stratification of both patient groups based on demographic characteristics including age range, sex, and region of residence. Our study was considered exempt from Institutional Review Board (IRB) approval due to the de-identification of the data prior to collection and analysis.

Google Scholar (2005-present) and PubMed (2005-present) were the primary search engines utilized during the literature review regarding the connection between CMV and malignancy. Studies of interest that were utilized in this paper mainly focused on how a latent CMV infection confers affected immunity against malignancy. Exclusion criteria included exempting studies done before the year 2000. The reference list of each included study was also heavily considered.

Keywords such as “bronchogenic carcinoma,” “human cytomegalovirus,” “neoplasia,” “tumor microenvironment,” and “oncomodulation” were utilized during this search to elucidate a possible connection between CMV and bronchogenic carcinoma.

## Results

**Figure 1:**
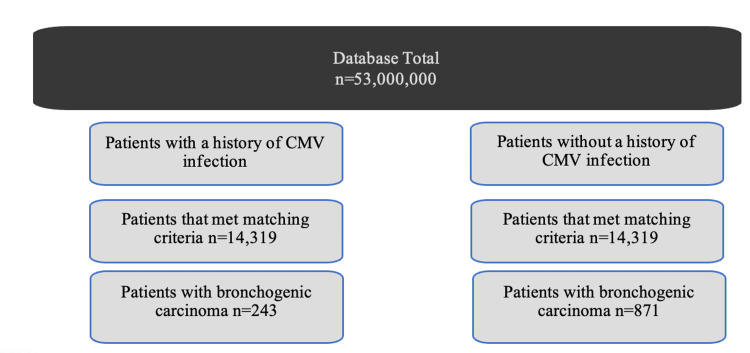
Diagram depicting how patients were grouped according to various criteria matched by age range and CCI score

**Figure 2:**
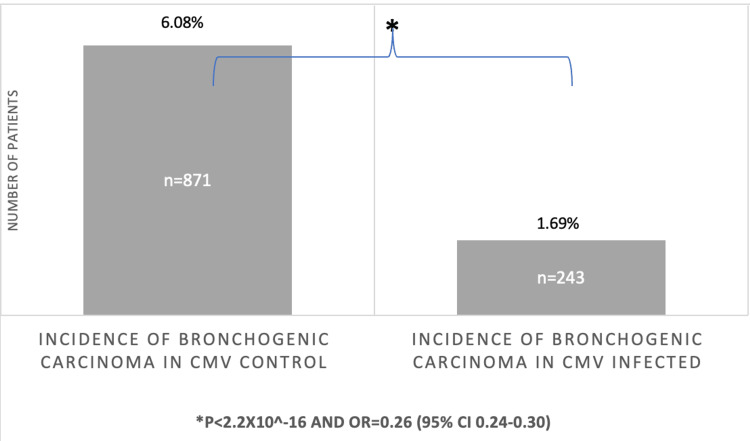
Incidence of bronchogenic carcinoma amongst individuals with and without a previous CMV infection matched by age range and CCI score

Prior CMV infection was associated with decreased incidence of developing bronchogenic carcinoma. Figure 1 illustrates the way the database was utilized to match both experimental and control groups by age range and CCI score. The incidence of bronchogenic carcinoma was 1.69% and 243 (out of 14,319) patients in the CMV group compared to 6.08% and 871 (out of 14,319) patients in the control group (Figure 2). The difference was statistically significant with a P-value of less than 2.6x10^-16^ and an odds ratio of 0.26 (95% CI [0.24-0.30]). The study also attempted to match the two groups for treatment. However, further evaluation of the outcomes being attributed to the treatment used for CMV in each group was limited due to the insufficient number of patients in the control group exposed to the CMV treatment.

**Figure 3:**
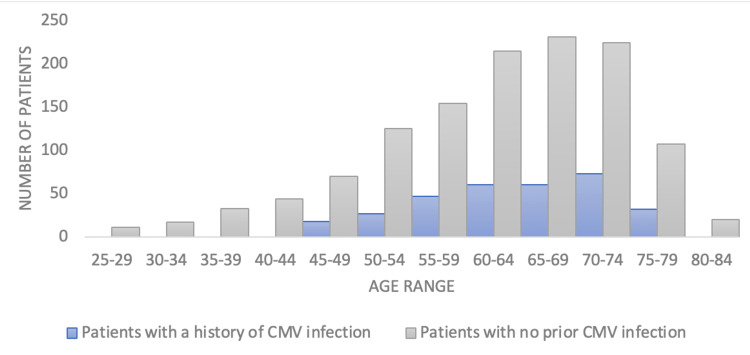
Age distribution of patients diagnosed with bronchogenic carcinoma with and without a history of CMV infection

**Figure 4:**
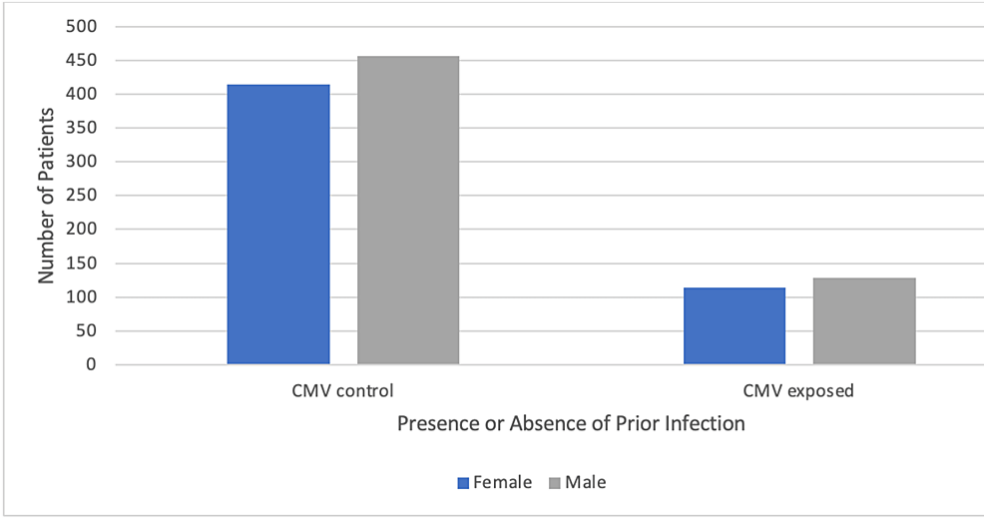
Gender distribution of patients affected with bronchogenic carcinoma amongst individuals with and without a previous CMV infection matched by age range and CCI score

The age at diagnosis of bronchogenic carcinoma was later in individuals with a prior CMV infection (Table 1). Additionally, the median age of diagnosis of lung cancer within the United States is 70 years old, aligning with what this study found to be the most prevalent age bracket at diagnosis which was 70-74 (Figure 3) [8]. The empty boxes found within table 1 indicate an insufficient patient population within those age brackets. Male patients had higher incidences of bronchogenic carcinoma in both the control and experimental populations studied within this database (Figure 4, Table 2).

**Table 1:** Age distribution of patients diagnosed with bronchogenic carcinoma with and without a history of CMV infection

Age Range	Patients without a history of CMV infection	Patients with a history of CMV infection
25-29	11	
30-34	17	
35-39	33	
40-44	44	
45-49	70	18
50-54	125	27
55-59	154	47
60-64	215	60
65-69	231	60
70-74	224	73
75-79	107	32
80-84	20	

**Table 2:** Sexes of patients with and without a history of CMV infection after being matched by age range and CCI score

	Patients without history of CMV infection	Patients with history of CMV infection
Males	456	129
Females	415	114

**Figure 5:**
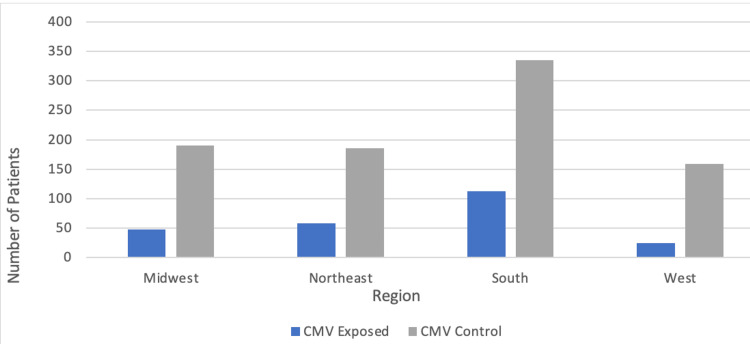
Regions of bronchogenic carcinoma development amongst individuals with and without a previous CMV infection matched by age range and CCI score

**Figure 6:**
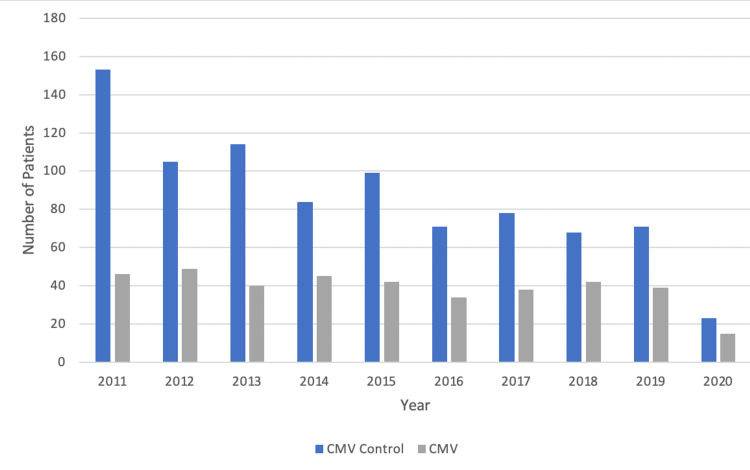
Prevalence in patients with bronchogenic carcinoma with and without a previous CMV infection matched by age range and CCI score

The region of development within the United States of bronchogenic carcinoma was found to be higher in the South amongst patients that had a previous CMV infection and patients with no prior history of a CMV infection (Figure 5). In the West, there was the lowest prevalence of bronchogenic carcinoma in both patient groups studied. The male sex was also found to have a higher number of patients in both the control and experimental groups (Table 2). The year 2011 had the highest number of patients diagnosed with bronchogenic carcinoma in the control CMV group, whereas the experimental CMV group had similar amounts of patients per year (Figure 6, Table 3). Both patient groups saw a considerable reduction of new diagnoses in the year 2020.

**Table 3:** Distribution of patients diagnosed with bronchogenic carcinoma by year matched by age range and CCI score

Year of Diagnosis	Patients without a history of CMV infection	Patients with a history of CMV infection
2011	153	46
2012	105	49
2013	114	40
2014	84	45
2015	99	42
2016	71	34
2017	78	38
2018	68	42
2019	71	39
2020	23	15

## Discussion

The study demonstrates the importance and needs to further investigate how CMV affects the growth of tumors [2]. An extensive evaluation is recommended to assess the specific immune mechanisms of CMV that lead to the reduction of bronchogenic carcinoma incidence and delay in diagnosis that was seen within this study. Potentially, the scientific data regarding possible therapeutic mechanisms outlined within this paper of a CMV infection may benefit patients who are at greater risk of developing bronchogenic carcinoma. This may be done by producing a stronger and more robust immune response against malignancy. Additionally, this study found that patients with a history of CMV infection had a later onset of bronchogenic carcinoma when compared to the control population, indicating the possibility of enhanced immuno-surveillance at an earlier point in life when compared to the control population (Figure 3) [9]. This is likely attributed to mechanisms in which CMV targets natural killer cells to induce the expansion of this cell line, which is due to systemic peripheral activation of the immune system, with this activation possibly lasting throughout a lifetime [10]. Additionally, the CMV control population had an earlier age of onset of bronchogenic carcinoma, indicating that a higher population of effector immune cells in the experimental group contributed to a more robust response against malignancy which delayed the onset of bronchogenic carcinoma. The vast increase in natural killer cells leads to more efficient killing of malignant cells through the release of perforins and granzymes and the formation of immune complexes [11]. With the expansion of natural killer cells, both the innate and adaptive immune system capacities within patients are vastly improved leading to the delay in the onset of bronchogenic carcinoma as seen in this study.

The tumor microenvironment is also greatly modulated by a latent CMV infection through the enhancement of inhibitory cytokines and the reduction of tumor cell extravasation [6]. Specifically, CMV activity against tumor cells may be pro-apoptotic with the utilization of caspases, thus altering and restricting tumor activity at the cellular level [6].

CMV has been postulated to induce caspases to stimulate apoptosis and to up-regulate Major Histone Compatibility (MHC) class II molecules potentiating an individual’s immune defenses against cancer [5]. These properties make the potential therapeutic use of this virus appealing to cancer researchers. However, current research details how CMV may be an oncogenic virus, with published studies detailing how breast cancer, colon cancer, liver cancer, cervical cancer, prostatic cancer, and malignant gliomas have been found to be positive for CMV, with CMV infection possibly suppressing host defenses in the tumor microenvironment and promoting carcinogenesis [12]. With these aspects in mind, the development of therapies utilizing CMV must be done judiciously as additional research is needed to further unfold the specific mechanisms behind how CMV impacts the immune system.

Potential limitations to this study include the inability of the database to adjust for all potentially confounding variables, including various patient co-morbidities and social history data that may increase the risk for cancer. Specifically, socioeconomic status and how this may influence one's exposure to carcinogens over time were not fully investigated. Moreover, patient information regarding occupational hazards or a genetic predisposition that could lead to a greater risk of developing lung malignancy was also not available. This information, if accessible, could have further strengthened the conclusions that this study found.

## Conclusions

Our study is the first to demonstrate that previous CMV infection may reduce the risk of developing bronchogenic carcinoma in certain populations. Future studies should focus on the exact mechanisms underlying the proliferation of natural killer cells in patients with a prior history of CMV infection. These studies should aim to further explain whether this induction of the immune system is a feasible therapeutic approach in the treatment and prevention of bronchogenic carcinoma.

Highlights

1. A history of CMV infection is associated with a decreased risk of developing bronchogenic carcinoma.

2. CMV is a ubiquitous herpes virus that induces long-lasting changes to the host immune system, which may confer increased protection against certain types of malignancy, while also dampening host immune responses against other neoplasms.

3. Further studies should focus on elucidating the etiology through which CMV-activated natural killer cells decrease the likelihood of developing bronchogenic carcinoma.
